# Designing a package of sexual and reproductive health and HIV outreach services to meet the heterogeneous preferences of young people in Malawi: results from a discrete choice experiment

**DOI:** 10.1186/s13561-015-0046-6

**Published:** 2015-05-09

**Authors:** Christine Michaels-Igbokwe, Mylene Lagarde, John Cairns, the Integra Initiative, Fern Terris-Prestholt

**Affiliations:** 1 London School of Hygiene and Tropical Medicine, Faculty of Public Health and Policy, Department of Global Health and Development, 15-17 Tavistock Place, London, WC1H 9SH UK; 2 London School of Hygiene and Tropical Medicine, Faculty of Public Health and Policy, Health Services Research and Policy, 15-17 Tavistock Place, London, WC1H 9SH UK

**Keywords:** Discrete choice experiment, Young people, Sexual and Reproductive Health, Family planning, HIV, Outreach

## Abstract

**Background:**

This article examines young people's preferences for integrated family planning (FP) and HIV services in rural Malawi. Different hypothetical configurations for outreach services are presented using a Discrete Choice Experiment (DCE). Responses are analysed using Random Parameters Logit and Generalised Mixed Logit (GMXL) models in preference space and a GMXL model parameterised in willingness-to-pay space. Simulations are used to estimate the proportion of respondents expected to choose different service packages as elements are varied individually and in combination.

**Results:**

Responses were collected from 537 young people aged 15–24. Results show that when considering attending an outreach service to access family planning young people value confidentiality and the availability of HIV services including HIV counselling and testing (HCT) and HIV treatment, though significant observable and unobservable heterogeneity is present. Female respondents and those aged 20–24 were less concerned with service confidentiality compared to male respondents and those aged 15–19; respondents who were in a relationship at the time of the survey valued confidentiality more than those who reported being single. The addition of sports and recreation for young people may also be an attractive feature of a youth-friendly service; however, preferences for this attribute vary according to respondent gender. Results of the simulation modelling indicate that the most preferred service package is one that offers confidential services, both HCT and HIV treatment and sports for youth, with up to 32% of respondents expected to choose this service over a service where clients may have concerns over confidentiality, only HCT is available and there are no additional activities for young people. Estimates of willingness-to-pay for service attributes indicate that respondents were willing to pay up to USD$1.76 for confidentiality, USD$0.65 for a service offering both HCT and HIV treatment and USD$0.26 for a service including sports for youth.

**Conclusions:**

Young people were able to complete a complex DCE and appeared to trade between the different characteristics used to describe the outreach services. These findings may offer important insight to policy makers designing youth friendly SRH outreach services and providers aiming to improve the acceptability and uptake of FP services.

**Electronic supplementary material:**

The online version of this article (doi:10.1186/s13561-015-0046-6) contains supplementary material, which is available to authorized users.

## Background

Uptake of family planning (FP) and HIV services among sexually active youth in sub-Saharan Africa remains low. High levels of unmet need for FP is associated with increased adolescent pregnancy leading to both increased morbidity and mortality [1-4]. There have been repeated calls to increase availability, accessibility and quality of sexual and reproductive health (SRH) services, including FP, for young people in low-income settings [4-7]. However, the evidence directly linking increased availability of youth-friendly services and increased uptake of FP services among youth is mixed [8,9] suggesting that there may be additional barriers to access or heterogeneity in youth preferences which may not have been addressed in the design and delivery of services for young people.

**Table 1:** **Final attributes and levels for DCE**

**Attributes**	**Levels**	**Justification**
Service Provider Gender	Male	Individuals in FGDs^d^ indicated that they may feel more comfortable with a same sex provider, but many indicated that providers of both genders should be available so that everyone would feel comfortable accessing the service
	Female	
Service Provider Age	Less than 30 years of age	Individuals in FGDs indicated that they may feel more comfortable with a younger or older service provider. No consensus came out in the discussion but individuals expressed strong preferences for one or the other.
	More than 30 years of age	
Availability of HIV services	HCT^a^ only	One of the project aims is to explore youth preferences for integrated SRH^e^ and HIV services. This attribute can provide insight into the value that youth place on the availability of HIV services in the context of an FP service regardless of whether they are likely to actually use these services.
	HCT and antiretroviral treatment available	
Confidentiality	You do not need to be concerned about confidentiality when you go for FP^b^. You can feel confident that the service provider will not share the details of your visit with anyone.	Youth reported concerns about confidentiality and talked about confidentiality in terms of both secrecy and anonymity. In the final questionnaire this attribute was described in relation to uncertainty over whether other clients may be within hearing distance when a client goes for a FP consultation and whether the client may feel confident that the service provider will not share any details of their visit.
	Sometimes when you go for FP other people are present or nearby. If there were other people present in the room or within hearing distance outside when you went for FP then you may feel the service is not private or confidential.	
Youth Focus	Recreation and sports activities are offered for young people when health workers come	In the qualitative work youth (especially boys) expressed a desire for recreation and sports activities and youth clubs.
	Music and drama with health messages are performed while health services are being offered	Previous outreach programmes available in the area included an aspect of health education or entertainment such as music and drama. These components of the outreach programme were recently discontinued due to funding constraints. They are included to explore how important these additional youth-friendly service components are to youth and whether their reintroduction may be valuable in attracting young people.
	Health education talks on issues important to youth are delivered before services begin	
	No additional activities (health services only)	
Price	Free	This range reflects prices that FGD participants mentioned paying in facilities in their catchment area. In the pilot a top price of 300MK was used but the results of the analysis suggested that some respondents were not responsive to price so the upper bound was increased to 500 MK.
	50 MK^c^	
	150 MK	
	500 MK	

^a^HCT = HIV Counselling and Testing, ^b^FP = Family Planning, ^c^MK = Malawi Kwacha (50MK was equal to approximately USD$0.20 in May-June 2012), ^d^FDG = Focus Group Discussion, ^e^SRH = Sexual and Reproductive Health.

**Table 2:** **Study population characteristics**

**Variable Name**	**Description**	**Study Sample (n)**	**Rural Areas in Malawi** ^**d**^
Age	15-19 years	60.7% (328)	56.3%
	20-24 years	39.3% (212)	43.7%
Gender	Male	50.2% (269)	48.2%
	Female	49.8% (271)	51.8%
Number of people normally resident in the household	1-4 people	44.4% (240)	55.3%
	5-8 people	48.5% (262)	40.0%
	9 or more	5.7% (31)	4.5%
	Missing	1.3% (7)	-
Average monthly household income	< MK^b^ 1,000/m	8.5% (46)	
	MK 1,000-4,999/m	44.6% (241)	
	MK 5,000-9,999/m	17.4% (94)	
	MK 10,000-19,999/m	4.1% (22)	
	MK 20,000-29,999/m	0.7% (4)	
	+ MK 30,000	0.7% (4)	
	Don't know	23.9% (129)	
Paid employment in the last 12 months	Yes	56.1% (303)	63%^e^
	No	43.9% (237)	37% ^e^
Employment Type	Employed full-time	2.6% (8)	
	Casual/Seasonal	65.3% (198)	
	Self-employed	23.1% (70)	
	Other	8.9% (27)	
School	Currently attending school	49.3% (266)	-
Relationship	Currently in a relationship	64.3% (347)	-
Sex	Sexually active in the past 12 months	77.6% (302)	-
FP^a^ Use, 15-19	Respondents aged 15–19 years who used a modern method of FP in the 12 months preceding the survey^c^	37.8% (124)	19.5%
FP Use, 20-24	Respondents aged 20–24 years who used a modern method of FP in the 12 months preceding the survey^c^	70.8% (150)	67.1%
Future FP	Non-users (n = 266) who indicated an intention to use FP in the future	91.1% (242)	-
Amount paid for FP at the last place accessed	Free	82% (244)	
	10 to 50MK	3% (9)	
	51 to 200MK	12% (35)	
	201 to 500MK	2% (5)	
	Can't remember	1% (3)	
N = 540			

^a^FP = Family Planning, ^b^MK = Malawi Kwacha (1000MK was equal to approximately USD$4.00 in May-June 2012), ^c^These figures are not directly comparable as this study measured FP use in the 12 months preceding the survey while the DHS data for rural Malawi represents ever use of FP, ^d^Source: National Statistical Office (2011), ^e^These figures are not directly comparable as DHS figures refer to employment status among both rural and urban residents.

**Table 3:** **Models I and II estimation of young people's preferences for integrated FP and HIV outreach services**

	**Model I (RPL)**				**Model II (RPL - Correlated)**			
	**Coefficient**	**SE** ^**a**^	**StdD** ^**a,b**^	**SE** ^**a,b**^	**Coefficient**	**SE** ^**a**^	**StdD** ^**a,b**^	**SE** ^**a,b**^
*Random Design Parameters*								
Provider Age (Older)	−0.053 **	0.025	0.183***	0.051	−0.167***	0.056	0.253***	0.042
Provider Gender (Female)	0.031	0.022	0.007	0.088	0.065	0.047	0.058	0.040
Confidential Service	1.328***	0.064	1.113***	0.062	1.417***	0.106	1.207***	0.066
HCT and ART Available	0.394***	0.034	0.499***	0.039	0.413***	0.076	0.533***	0.051
Sports for Youth	0.235***	0.047	0.123	0.142	0.541***	0.107	0.268***	0.084
Music and Drama	−0.032	0.052	0.403***	0.065	−0.172	0.118	0.503***	0.076
Health Talk	0.074	0.047	0.044	0.134	0.294***	0.110	0.331***	0.075
Price	−0.003***	0.000	0.004***	0.000	−0.004***	0.001	0.005***	0.000
*Fixed Parameter*								
Alternative Specific Constant	−2.417***	0.071			−2.458***	0.078		
*Interaction Terms*								
Prov. Older: Resp. Female					0.048	0.055		
Prov. Older: Resp. Older					−0.036	0.056		
Prov. Older: In Relationship					0.017	0.057		
Prov. Female: Resp. Female					−0.036	0.047		
Prov. Female: Resp. Older					0.056	0.049		
Prov. Female : In Relationship					−0.053	0.049		
Confidentiality: Resp. Female					−0.412***	0.106		
Confidentiality: Resp. Older					−0.029	0.118		
Confidentiality: In Relationship					0.474***	0.112		
HIV Services: Resp. Female					0.090	0.071		
HIV Services: Resp. Older					−0.142*	0.078		
HIV Services: In Relationship					0.141*	0.074		
Sports: Resp. Female					−0.350***	0.103		
Sports: Resp. Older					0.194*	0.105		
Sports: In Relationship					−0.137	0.105		
Music: Resp. Female					0.065	0.116		
Music: Resp. Older					−0.008	0.118		
Music: In Relationship					0.180	0.118		
Health Talk: Resp. Female					−0.095	0.106		
Health Talk: Resp. Older					−0.146	0.112		
Health Talk: In Relationship					−0.078	0.111		
Price: Resp. Female					0.000	0.001		
Price: Resp. Older					0.001	0.001		
Price: In Relationship					0.000	0.001		
Variance parameter in scale (τ)								
Weighting parameter (γ)								
Sample mean (σ)								
*Model Fit Statistics*								
Number of individuals	537				537			
Number of observations	6444				6444			
Log Likelihood Function	−3578				−3501			
AIC	7191				7139			
% of responses correctly predicted	62%				62%			

^a^SE = Standard Error, StdD = Standard Deviation, ^b^Only for random parameters.

***p < 0.01; **p < 0.05; *p < 0.1.

Likelihood Ratio Test Between Models 2 and 1: LR_2–1_ = 156.66χ^2^_52.0.0.001_(89.27).

**Table 4:** **Model III and Model IV estimation of young people's preferences for integrated FP and HIV outreach services**

	**Model III (GMXL-Correlated)**				**Model IV (GMXL WTP-Space)**			
	**Coefficient**	**SE** ^**a**^	**StdD** ^**a,b**^	**SE** ^**a,b**^	**Coefficient**	**SE** ^**a**^	**StdD** ^**a,b**^	**SE** ^**a,b**^
*Random Design Parameters*								
Provider Age (Older)	−0.228**	0.111	0.198*	0.104	−17.45	14.25	7.98	10.48
Provider Gender (Female)	0.146	0.101	0.034	0.082	−16.18	16.30	20.93	14.81
Confidential Service	2.609***	0.275	2.042***	0.118	441.10***	47.83	309.80***	29.56
HCT and ART Available	0.611***	0.146	0.844***	0.096	161.36***	25.927	163.70***	20.47
Sports for Youth	0.952***	0.267	0.655***	0.165	65.27*	35.40	60.07*	35.90
Music and Drama	−0.282	0.260	0.711***	0.209	−6.43	39.55	59.33*	31.76
Health Talk	0.395*	0.238	0.622***	0.226	60.30	36.82	52.06**	21.56
Price	−0.006***	0.001	0.008***	0.001				
Price in preference space form					−0.01***	0.002	0.01***	0.001
*Fixed Parameter*								
Alternative Specific Constant	−2.529***	0.059			−2.67***	0.067		
Price in WTP-Space					1			
*Interaction Terms*								
Prov. Older: Resp. Female	0.088	0.115			13.34	16.20		
Prov. Older: Resp. Older	−0.095	0.124			−8.43	18.13		
Prov. Older: In Relationship	−0.070	0.118			7.45	16.50		
Prov. Female: Resp. Female	−0.098	0.106			−18.91	15.64		
Prov. Female: Resp. Older	−0.011	0.103			−1.26	15.47		
Prov. Female : In Relationship	0.060	0.107			7.05	17.07		
Confidentiality: Resp. Female	−0.476***	0.179			−4.45	21.41		
Confidentiality: Resp. Older	−0.317*	0.172			−15.18	20.26		
Confidentiality: In Relationship	0.850***	0.204			57.14**	23.64		
HIV Services: Resp. Female	0.160	0.141			26.28	25.13		
HIV Services: Resp. Older	−0.285**	0.144			−9.04	24.41		
HIV Services: In Relationship	0.416***	0.159			4.68	25.54		
Sports: Resp. Female	−0.424*	0.233			−25.85	25.44		
Sports: Resp. Older	0.282	0.254			25.92	33.24		
Sports: In Relationship	−0.092	0.257			−15.47	29.60		
Music: Resp. Female	0.146	0.262			16.67	39.14		
Music: Resp. Older	−0.278	0.296			−14.87	43.58		
Music: In Relationship	0.212	0.252			19.93	40.58		
Health Talk: Resp. Female	−0.220	0.252			16.45	39.99		
Health Talk: Resp. Older	−0.164	0.261			−12.81	41.12		
Health Talk: In Relationship	−0.110	0.257			−6.37	43.69		
Price: Resp. Female	−0.001	0.001			−0.17	0.11		
Price: Resp. Older	0.001	0.001			−0.03	0.10		
Price: In Relationship	0.002**	0.001			−0.01	0.12		
Variance parameter in scale (τ)	0.997***	0.067			1.37***	0.10		
Weighting parameter (γ)	0.00041	0.032			0			
Sample mean (σ)	0.976	1.161			0.95	1.78		
*Model Fit Statistics*								
Number of individuals	537				537			
Number of observations	6444				6444			
Log Likelihood Function	−3477				−3530			
AIC	7096				7187			
% of responses correctly predicted	62%				62%			

^a^SE = Standard Error, StdD = Standard Deviation, ^b^Only for random parameters.

***p < 0.01; **p < 0.05; *p < 0.1.

Likelihood Ratio Test Between Models 3 and 2: LR_3–2_ = 47.01χ^2^_2.0.0.001_(13.82).

Likelihood Ratio Test Between Models 4 and 3: LR_4–3_ = 107.19χ^2^_8.0.0.001_(26.125).

**Table 5:** **WTP for Service Attributes Estimated in WTP-Space (2011 USD)**

	**Mean**	**Std Dev.**
Provider Age (Older)	−0.07	0.03
Provider Gender (Female)	−0.06	0.08
Confidential Service	1.76***	1.24
HCT and ART Available	0.65***	0.65
Sports for Youth	0.26*	0.26
Music and Drama	−0.03	0.24
Health Talk	0.24	0.21

**Table 6:** **Simulated service package scenarios**

**Scenario**	**Service package elements**	**Comparison scenario**	**Base case scenario**
1	1	Change price from free to 50MK*	Older, female provider, confidentiality, HCT and ART available, sports
2	2	Change price from 50MK to 150MK	
3	3	Change Price from 150MK to 500MK	
4	4	Add sports	Older, female provider, confidentiality, HCT and ART available, no youth friendly component, free
5	5	Add health talk	
6	6	Add confidentiality	Older, female provider, uncertainty about confidentiality, HCT and ART available, no youth friendly component, free
7	7	Add HIV services	Older, female provider, confidentiality, HCT only available, no youth friendly component, free
8	8	Younger service provider	Older, female provider, confidentiality, HCT and ART available, no youth friendly component, free
9	4 and 6	Add sports and confidentiality	Older, female provider, uncertainty about confidentiality, HCT and ART available, no youth friendly component, free
10	5 and 6	Add health talk and confidentiality	
11	6 and 7	Add confidentiality and HIV Services	Older, female provider, uncertainty about confidentiality, HCT only available, no youth friendly component, free
12	4, 6 and 7	Add sports, confidentiality and HIV services	
13	5, 6 and 7	Add health talk, confidentiality and HIV services	
14	1, 4 and 6	Price change from free to 50MK with sports and confidentiality	Older, female provider, uncertainty about confidentiality, HCT and ART available, no youth friendly component, free
15	1, 5 and 6	Price change from free to 50MK with HIV services and confidentiality	Older, female provider, uncertainty about confidentiality, HCT only available, no youth friendly component, free
16	1, 4, 5 and 6	Price change from free to 50MK with confidentiality, sports and HIV services	

*MK = Malawi Kwacha, 50MK was equal to approximately USD$0.20 in May-June 2012.

**Table 7:** **Proportion of respondents choosing simulated service package scenarios compared to base case (%)**

**Scenario**	**Full Sample**	**Female**	**Male**	**Age 15–19 years**	**Age 20–24 years**	**In a relationship**	**Not in a relationship**
	***(n = 537)***	***(n = 268)***	***(n = 268)***	***(n = 328)***	***(n = 209)***	***(n = 343)***	***(n = 194)***
1	−4.1	−3.6	−4.4	−4	−4.1	−3.8	−4.6
2	−6.4	−5.6	−7.1	−6.3	−6.5	−5.9	−7.1
3	−11.9	−10.5	−13.4	−11.8	−12.2	−11.2	−13.2
4	12.1	14.4	9.7	14.3	8.6	8.6	18.1
5	5.4	2.8	7.9	6.7	3.2	3.8	8.2
6	21.3	21.1	21.5	19.7	23.8	20	23.7
7	13.2	11.7	14.6	11.8	15.3	14	11.7
8	4.7	5.2	4.2	5.4	4.1	4.2	5.6
9	26.5	27.1	25.8	16.8	21.2	24.4	30.2
10	22.3	21.4	23.3	15.6	20.1	20.7	25.3
11	25.4	24.7	26.1	25.8	27.4	24.7	26.7
12	29.8	29.8	29.7	28.9	31.1	28.4	32.2
13	26	24.7	27.2	24.6	28	24.9	27.8
14	25.6	26.3	24.8	24.9	26.6	23.4	29.4
15	24.6	23.9	25.2	22.8	27.4	23.9	25.8
16	29.1	29.2	29	28.2	30.5	27.7	31.6

In addition to the development of youth-friendly services, recent efforts to improve access to FP and HIV services in low- and middle-income countries have focused on service integration. At the operational level, the term integration may include efforts to join FP and HIV services through the coordination of financing and policy. At the service delivery and facility levels, integration may refer to methods of coordinating service delivery to allow individuals to access multiple services in a single consultation, either by the same provider or on the same site through a system of internal referral [10]. Both types of integration have the potential to improve access to FP services, reduce stigma associated with accessing HIV services and increase patient satisfaction. However, available evidence to date shows that the extent to which this potential is realised through service delivery and facility level integration is mixed; missed opportunities to provide multiple services in a single visit persist [11-16], and the complex issues that surround stigma and patient satisfaction are not adequately addressed in many integrated service delivery configurations [15,17,18]. Resource constraints, high patient volumes and inadequate provider training contribute to difficulties in effectively integrating services, but despite these challenges there is consensus among patients and policy makers alike regarding the need to better integrate FP and HIV service delivery and to understand how to design services that are responsive to client needs [14,19].

In order to develop a patient-centred approach to the design of integrated FP and HIV services for young people it is important to identify which aspects of service delivery youth value, to understand the relative importance of service characteristics and to understand how preferences are likely to vary among different groups. To date, no empirical work has assessed patient preferences for integrated FP and HIV services or examined the impact of the service package configuration on young people’s preferences. We used a discrete choice experiment (DCE) to assess young people’s stated preferences for the design and delivery of integrated FP and HIV outreach services in rural Malawi. The research was conducted in partnership with the Family Planning Association of Malawi (FPAM), a local non-governmental organisation providing both facility based and outreach services in four districts in Malawi. In line with the government focus on improving access to SRH and HIV services to young people, FPAM is working to expand outreach service provision including the delivery of FP and HIV counselling and testing (HCT) in the four districts it is currently operating in. The present study was designed to inform the design and delivery of outreach services though this expansion.

The aim of this research was to identify the relative importance of service characteristics; to identify additional youth-friendly service components that may be attractive for young people; to understand preferences for integrated FP and HIV service delivery; to explore heterogeneity in youth preferences; and to estimate willingness-to-pay (WTP) for service attributes. It was hypothesised: (i) that male and female respondents would have different preferences for service attributes, (ii) that younger respondents would have less well formed preferences and that this would be revealed through heterogeneity in preferences for service attributes^a^ and (iii) that individuals who have experience in using FP or are currently in a relationship would have different preferences for family planning services.

DCEs are a multi-attribute survey method used to indirectly elicit consumer preferences by presenting respondents with hypothetical situations which may be seen as reasonable substitutes and asking them to choose between alternatives [20]. Using this approach, a health service may be described by a number of characteristics or attributes. Choice profiles are then created with varying levels of each attribute and paired or grouped to create choice sets. Respondents are asked to make repeated choices over a number of different choice sets in order to observe the tradeoffs that they make as the levels of each attribute varies. The value that respondents place on each of the good’s attributes is inferred by calculating the marginal rate of substitution between attributes [21]. Where price is included as an attribute, WTP for a change in attribute levels may be estimated as the negative ratio of the attribute and price parameters.

In addition to allowing for the exploration of preferences for services which are currently not available, DCEs may include attribute levels that are outside the range of those that patients normally encounter [22]. This can be helpful for understanding the impact of changes to current services and may be helpful in identifying strategies to make services more attractive to clients. Such analyses can inform the design and delivery of services or interventions and may be helpful in identifying strategies to increase the acceptability or uptake of existing or new services.

DCEs are commonly used in health economics to elicit patient preferences for a variety of services and health care delivery models [23-26]. They have also been used to explore preferences for and predict uptake of existing and new products [27-29]. The majority of published DCEs in health economics have been conducted in high income settings and with adult populations [30]. Few examples of DCEs done with young people have been published and these were conducted in high income settings [27,31,32]. As a result, little is known about the use of DCEs among young people in low- and middle-income settings. This study provides the first example of a DCE conducted among young people in a low-income setting.

## Methods

### DCE development

A literature review of reported barriers and facilitators to accessing FP and HIV services was used as a starting point for identifying attributes that could be included in the DCE. This was followed by a choice mapping process which helped to define the decision problem that the DCE would focus on [33]. These two steps helped to inform the design of topic guides and questionnaires used in 12 focus group discussions (FGDs) and three key informant interviews (KIIs) conducted with young people aged 15–24 in three communities in Ntcheu District, Malawi. (Details of the qualitative work are available in [33] and [34]). These were conducted in Chichewa by locally trained facilitators and transcribed and translated into English. Transcripts were coded and analysed using a thematic approach whereby data were categorised and summarised according to themes identified through the coding and analysis process [35]. In general, respondents were aware of outreach services provided in one of the research communities and described outreach services as offering family planning HCT and immunisation services. Concerns about confidentiality in existing services were paramount in all FGDs and price was considered a major barrier to accessing services. Views on the age and gender of an ideal service provider were varied. Some respondents expressed concern over infrequent or irregular service provision. However, respondents indicated that having services available in their community would be beneficial in that it would reduce the amount of travel required to access services [33].

### Choice of the attributes and levels

The qualitative work identified service delivery attributes likely to influence young people’s choice of outreach service, namely: service provider characteristics such as age, gender, training and attitude; the package of services available; the availability of special activities for youth; confidentiality of the service; waiting time and price. This list was narrowed to include those factors expected to account for the majority of variation in choice and those that could be influenced by policy changes to improve the design of services. Attributes that could be influenced by policy were identified in discussion with FPAM, who was engaged in outreach service provision in Ntcheu District at the time of the survey. The focus of this discussion was to identify aspects of service delivery that could realistically be offered in the short and long term and to structure the choice sets in a way that reflected current service delivery in other areas of Ntcheu District.

Attributes included in the final DCE were: age and gender of service provider; confidentiality; availability of HIV services; special youth friendly service components and price. The HIV services attribute included two levels: ‘HIV Testing and Counselling (HCT) is available at the outreach’ and ‘HCT and Antiretroviral Therapy (ART), the treatment for HIV is available at the outreach’. Youth friendly components included sports for youth, music and drama and health talks.

Levels were selected in part based on the categories and language used by participants in the qualitative research and in part based on practicality. For example, only two levels were included for the HIV services attribute in part because HCT is routinely offered by FPAM in outreach settings and all health service facilities identified by respondents in the qualitative work offered HCT. Further given the government focus on both integrating HIV and SRH services and expanding access to HCT in all settings, it was assumed that all outreach services would provide some form of HCT in the future. Thus, including a third level for this attribute with no HCT would not provide a realistic picture of the likely structure of outreach services to be offered in the area. Additionally, since all other attributes had two or four levels, having one attribute with three levels would have impacted level balance and expanded the experimental design, adding to the number of tasks that each respondent would be required to answer. A larger experimental design could have been accommodated by blocking, but as a second blocked DCE was included on the overall survey, this would have presented logistical challenges in the field administration of the survey and so the decision was taken to limit the levels for all attributes to either two or four. A list of the final attributes and levels is provided in Table 1 along with a justification for the selection of the levels.

### Survey construction

**Figure 1:**
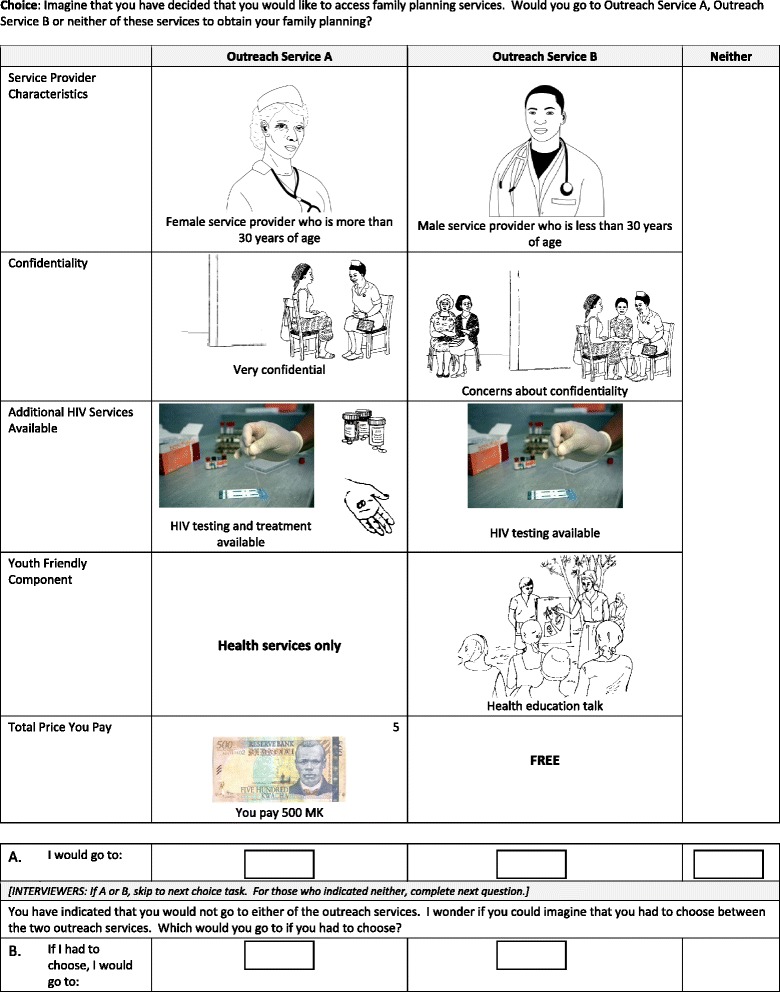
Sample choice task with images.

Pictures were used to represent the levels of each attribute to assist respondents with low levels of literacy. The images were refined following feedback from the pilot survey. A sample choice task from the final survey is presented in Figure 1

. The final DCE questionnaire included a description of each of the images which was read to participants prior to the start of the choice tasks and participants were invited to ask questions at any time to clarify the meaning of the images. An evaluation survey among 31 participants confirmed that respondents were able to correctly identify and describe individual images more than 75% of the time and 97% of respondents were able to correctly identify the difference between two images representing different levels of individual attributes.

### Framing the choice task

Given the generally low levels of uptake among youth in many low income settings, it was expected *a priori* that many respondents would not have experience in using FP services. However, since the aim of the research was to identify aspects of service design that are attractive to individuals not currently using FP as well as current FP users, it was important to ensure that the preferences of both groups were captured in the DCE. Individuals may not use FP services either because (i) they have no demand for FP or (ii) there are barriers to service use or no suitable services are available. Strategies to increase uptake that are specifically related to the design and configuration of services address the preferences latter group. As increasing uptake of FP services is a particular concern in this setting it was important to ensure that the preferences of both FP users and non-users were included in order to ensure that strategies to improve the acceptability of services for all youth could be identified.

If participants were asked to indicate which service they would like to use *if* they wanted to use FP services then ‘neither’ responses could be interpreted either as a choice not to use FP or not to use any service at all. This would obscure the preferences of individuals who would like to use a service, but who find the alternatives presented are not suitable. In order to ensure that the preferences of both FP users and non-users preferences were considered, and to ensure that ‘neither’ responses were comparable, the choice task was framed as asking participants to imagine that they have already decided to use FP services, and then indicate which of the services presented in the choice task they would use.

Additional survey questions included socio demographic characteristics (SDCs), knowledge of FP and HIV, FP use in the 12 months preceding the survey and future FP intentions. Data on FP use in the 12 months preceding the survey were intended to be used to split the sample for analysis in the event that preferences varied significantly between individuals who had experience in accessing FP services and those who did not.

### Experimental design

The experimental design for the unlabelled DCE with two alternatives and an opt-out was generated using Ngene [36]. The six attributes required a minimum of 12 choices. A D-efficient^b^ design was initially developed for the pilot using zero priors for all attributes. This design was then piloted with 40 young people aged 15–24 whose responses were analysed with both Multinomial Logit (MNL) and Random Parameters Logit (RPL) models. The final design was developed using parameter estimates from the RPL analysis which were incorporated as Bayesian priors with a normal distribution. Designs were developed for both an opt-out and a forced choice format and the final design averaged over both specifications so that the D-error would be minimised for both approaches^c^. The final unforced choice design had a median D-error estimate of 0.065. Results presented here are based on unforced choice data only.

### Final sample and survey implementation

The final survey was conducted in May – June 2012 in one Traditional Authority in Ntechu District, Malawi consisting of 15 villages. Seven villages were randomly selected to participate. Of these none had a formal or permanent health facility and one had intermittent outreach services. A sampling frame, developed through a community mapping exercise, identified 910 youth between the ages of 15 and 24 (the target population for the research) in the participating communities. From this list 620 individuals were randomly selected and invited to participate^d^. A minimum of three contacts were attempted for each individual and names replaced for those that could not be traced. No individuals contacted declined to participate. The final sample included 537 completed questionnaires.

### Framework for analysis of youth preferences

Discrete choice theory is rooted in Lancaster’s Theory of Demand which assumes that: consumers seek to maximise their utility by consuming goods; that goods are selected based on the combination of attributes contained within the goods; and that consumer preferences can be observed based on consumption choices [37]. This approach to consumer demand is rooted in random utility theory which assumes that consumer choices are probabilistic rather than deterministic. Generally, utility from a given outreach service can be expressed as [22]:

1 $$ {U}_{ji}={V}_{ji}+{\varepsilon}_{ji}=\alpha +{\beta}_1{X}_{1ji}+\cdots +{\beta}_{kj}{X}_{kji}+{\beta}_z{Z}_i+{\varepsilon}_{ji} $$

Where *U*_*ni*_ is the utility or satisfaction obtained by individual *i* from choosing alternative *j* of the alternatives *j* = 1, . . . , *j, . . .,J* available in the choice set described by *k* service attributes. The vector *V*_*ji*_ is made up of *β*_*j*_ *X*_*ji*_, where *X*_*ji*_ are the observable outreach service attributes and *β*_*j*_ are the coefficient estimates for each attribute. Observable individual characteristics *β*_*z*_ *Z*_*i*_, where *Z*_*i*_ are individual characteristics and *β*_*z*_ represents the relative influence of these characteristics on choice. All other unobservable characteristics are captured in *ε*_*ji*_, an error term incorporating all other unexplained variation such as heterogeneity in tastes across individuals and errors in measurement or model specification and is assumed to be independently and identically distributed (IID).

Individuals are assumed to choose the service associated with the highest utility. Thus, the probability that individual *i* chooses service *j* over service *n* is given as:

2 $$ P{r}_{ji}= Pr\left({U}_{ji}>{U}_{ni}\right) = \Pr \left({V}_{ji} - {V}_{ni}>\kern0.5em {\varepsilon}_{ji} - {\varepsilon}_{ni}\right)=\kern0.5em  \Pr \left({V}_{ji}+{\varepsilon}_{ji} > {V}_{ni}+{\varepsilon}_{ni}\right) $$

This basic approach is extended to the RPL model in which the error component is decomposed into two parts:

3 $$ {U}_{ji} = {V}_{ji} + \left[{\eta}_{ji} + {\varepsilon}_{ji}\right] $$

In this case, instead of estimating population level coefficients as above, individual level coefficients are estimated as follows:

4 $$ {\beta}_{ik} = {\beta}_k+{\delta}_k{Z}_i + {\Gamma}_k{v}_{ik} $$

Where observed heterogeneity is reflected in *δ*_*k*_ *Z*_*i*_ and unobserved heterogeneity is reflected in Γ_*k*_ *v*_*i*_ [38]. This model can accommodate heterogeneity in observable attributes through the random component *η*_*ji*_ which follows a distribution specified by the analyst. This allows for similarities in choice strategy across individuals. This is appropriate in the present analysis given that each respondent provided 12 observations. The remaining unobservable error component *ε*_*ji*_ is assumed to be IID.

The GMXL model is an expansion of the RPL model and offers a more flexible approach to identifying heterogeneity in the means of coefficients as well as in the error, or scale component [39]. The scale component represents unobservable but systematic variation in respondent choice. This may be a reflection of decision heuristics, such as lexicographic preferences, or of ‘randomness’ in respondent behaviour, all of which may be associated with choice task complexity or fatigue [39,40]. The model is given by:

5 $$ {\beta}_{ik} = {\sigma}_i\left({\beta}_k+{\delta}_k{Z}_i\right)+\left(\gamma + {\sigma}_i\left(1 - \gamma \right)\right)\Gamma {v}_i $$

The weighting parameter *γ* shows how variance in residual preference heterogeneity may vary with scale. The additional individual specific random scale factor *σ*_*i*_ is defined as:

6 $$ {\sigma}_i= \exp \left[\overline{\sigma} + {\updelta}^{\hbox{'}}{\mathrm{h}}_i + \tau {w}_i\right] $$

Here h_*i*_ represents observed heterogeneity (which may overlap with *Z*_*i*_) with a coefficient of δ and *w*_*i*_ represents unobserved heterogeneity with a coefficient of *τ* [38].

In the context of choice modelling willingness-to-pay may be estimated as the ratio of the marginal utility of one attribute and the negative marginal disutility of price.

7 $$ WTP = - \frac{marginal\  utility\  of\  attribute}{marginal\  disutility\  of\  price} $$

Using the RPL and GMXL approaches the price parameter may be specified as fixed or random. Where the price parameter is specified as random it is allowed to follow a distribution specified by the analyst. Where other attributes are also specified as random, calculation of WTP will involve computing the ratio of two randomly distributed terms. Depending on the choice of distribution, the resulting distribution of WTP estimates may not be defined or may produce unrealistic results [41]. An alternative approach is to estimate the model in terms of WTP [42]. This involves reparameterising the model so that instead of separately estimating coefficients for each attribute, with assumptions about the distribution of each coefficient, the ratio of the attribute and price is estimated directly [41]. This approach is referred to as estimation in *WTP-space* and can be contrasted to traditional approaches of estimating coefficients individually which is referred to as estimation in *preference space* [42]. Extending the GMXL model to WTP-space, coefficients are estimated as [38]:

8 $$ {\beta}_i = {\sigma}_i{\beta}_c\left[\frac{1}{\left(\frac{1}{\beta_c}\right)\left(\beta + \Gamma {v}_i\right)}\right]={\sigma}_i{\beta}_c\left[\frac{1}{\theta_c + \Gamma {v}_i}\right]\cdot $$

A particular advantage of estimation in WTP-space is that variation in WTP estimates are separated from variation in the price parameter, resulting in lower standard deviations and eliminating extreme values in the distribution which would seem to imply that some individuals are willing to pay very high amounts for individual attributes [42].

### Econometric specifications

Four models were estimated to assess respondent preferences. Models I is an RPL model with no correlation in parameters and no interaction terms. Model II in an RPL model with correlated parameters and interactions with SDC variables. Model III is a GMXL model with correlation and interaction terms. Models I through III are estimated in preference space and Model IV is an extension of Model III estimated in WTP-space.

All service attributes except price were effects coded^e^ in order to avoid confounding with the mean. The variable describing different youth friendly components had four levels and these were made into three effects coded variables interpreted relative to the base category of ‘no additional youth friendly component’. The price variable was coded according to the original values in Malawi Kwacha: 0, 50, 150 and 500 (equal to US$0, $0.20, $0.60 and $2.00 at the time of the survey in May-June 2012).

Observable heterogeneity in preferences was incorporated into the model through the use of SDC variables. These variables were interacted with service attributes in order to identify differences in preferences according to observable participant characteristics. Final SDC variables included participant gender, age category (15–19 and 20–24 years of age) and relationship status. The hypothesis relating to the inclusion of relationship status or previous experience using FP as SDCs was intended to capture heterogeneity in preferences among these sub-groups relative to the overall sample. Exploratory analysis revealed these variables to be highly correlated and so there was limited benefit to including both variables in the model. Separate models were developed including both of these variables as SDCs and each of them separately. No statistically significant variation in preferences was revealed in either of the models including previous experience using FP as an SDC and so only relationship status was selected for inclusion in the final model. This is not unexpected since this variable allows for broader exploration of variation in preferences according to intention to use FP as well as the choice not to use FP within the context of a relationship rather than restricting to previous experience only. SDCs were dummy coded with male, 15–19 years of age and not in a relationship as the respective base categories.

All of the models were described according to three utility functions, one for each of the two outreach services and one for the opt-out alternative.

$$ {U}_{Outreach\ A},\ {U}_{Outreach\ B} = \beta 1 Provider\  Age + \beta 2 Provider\  Gender + \beta 3 Confidential + \beta 4 HIVART + \beta 5 Sport + \beta 6 Music + \beta 7 HTalk + \beta 8 Price $$

9 $$ {U}_{None}=AS{C}_{None} $$

All parameters are generic across the two outreach services and the opt-out alternative contains only an alternative specific constant (ASC). Interaction effects were added as additional parameters to this base model.

The model was estimated using a maximum likelihood approach with simulations based on 500 Halton draws and start values were obtained from the corresponding MNL model. All service attributes were specified as random and parameters were set to follow a normal distribution. Model fit was compared using log likelihood ratio (LLR) tests, Akaike’s Information Criteria (AIC)^f^ and the proportion of correctly predicted responses. All of the models were estimated using NLOGIT 5 [43].

### Simulating young people’s preferences for package configurations

The results of Model III were used to investigate the impact of a change in service attributes on preferences. This was achieved by calculating the probability that a respondent would choose the new simulated service over a base case scenario.

$$ \Pr (sim)=P\left({U}_{sim}>{U}_{base}\right) = \frac{e^{U_{sim}}}{e^{U_{sim}}+{e}^{U_{base}}} $$

and

10 $$ \Pr (base)=P\left({U}_{base}>{U}_{sim}\right) = \frac{e^{U_{base}}}{e^{U_{sim}}+{e}^{U_{base}}} $$

Two sets of scenarios were specified. The first set of eight simulations involved changing attributes one at a time. In these scenarios the change in probability of respondents choosing the simulated scenario over the base case corresponds to the utility associated with the new level of the attribute. The second set included eight different simulated service packages designed to evaluate the combined effect of changes in more than one attribute. For these scenarios reported changes correspond to the overall utility associated with each simulated service package^g^. In addition to estimating changes in preferences for service packages for the full sample, simulations were stratified by the SDC variables used in the main analysis.

Ethical approval for the study was obtained from the Research Ethics Committees of the University of Malawi, College of Medicine and the London School of Hygiene and Tropical Medicine.

## Results

### Sample characteristics

A description of the study population is provided in Table 2. Selected sample characteristics are compared to those of the general population in Malawi using the results of the 2010 Demographic and Health Survey (DHS) [44] in order to assess generalisability of results. Comparable data on FP use were not available since the since respondents in the present study were asked about FP use in the past 12 months and the DHS sample reports ever use of FP which is a cumulative estimate of FP use over a respondent’s lifetime. According to the 2010 DHS, among young people living in rural areas of Malawi aged 15–19 and 20–24 years, 19.5% and 67.1% had ever used a modern method of contraception respectively. In the study sample, 37.8% of respondents aged 15–19 years and 70.8% of respondents aged 20–24 years had used FP in the past year.

### Young people’s preferences for integrated FP and HIV outreach services

The four models are broadly similar in terms of the sign and statistical significance of the coefficients of the design parameters (see Table 3 and 4). The models with correlated parameters pick up slightly more heterogeneity as shown in the statistical significance of the standard deviation of the design parameters. The statistically significant diagonal values in the Cholesky matrix highlight correlation between attributes making an uncorrelated specification inappropriate (matrices for models with correlation available in Appendix A). The LLR tests indicate that in terms of fit Model II is superior to Model I (*p* < 0.001), Model III is superior to Model II (*p* < 0.001) and Model IV is superior to Model III (*p* < 0.001). Model III has the lowest AIC value, confirming that this model provides the best fit to the data as estimated in preference space, and thus these results were used evaluate preferences for simulated service package scenarios. The main results presented below are based on Model III with WTP estimates derived from the WTP-Space model and simulation results presented subsequently. WTP estimates derived from Models II and III are presented in Appendix B. Estimates derived from the WTP-Space model are preferred as this approach provides more behaviourally plausible estimates with considerably lower standard deviations.

The coefficients for six of the eight design variables are statistically significant: confidentiality (β = 2.609), sports for youth (β = 0.952), availability of HCT and ART (β = 0.611), health talks (β = 0.395), provider age (β = −0.228) and price (β = −0.006). Of these coefficients all except provider age and price have a positive sign indicating that respondents are more likely to choose a service which is confidential, provides both HCT and ART, has sports for youth or includes a health talk. Respondents are less likely to choose a service with an older service provider (more than 30 years of age) and prefer lower priced services. The alternative specific constant for the None alternative is negative and statistically significant (β = −2.529) indicating that the service alternatives were generally acceptable to respondents and were preferred over the None alternative. The gender of the service provider and the availability of music and drama as a youth-friendly service component did not appear to influence respondent choices.

### Heterogeneity in preferences

Observable heterogeneity around the mean coefficient estimates for service attributes is revealed through the introduction of interactions between these parameters and SDC variables. The significance of the interaction terms confirms in part the hypotheses that preferences would vary according to participant gender and relationship status. The majority of the statistically significant heterogeneity is seen around the mean parameter estimates for confidentiality and HIV services where variation was seen according to more than one of the SDC variables. While some differences in preferences according to age are apparent, these are not systematic enough across all service attributes to suggest that younger respondents categorically had different preferences.

The coefficients of the interaction terms indicate that female respondents value confidentiality less than their male counterparts (*β = −*0.476), that older respondents value confidentiality less than younger respondents (*β = −*0.317) and that individuals who are currently in a relationship value confidentiality more than those that are single (*β =* 0.850).

When considering additional HIV services, older respondents (aged 20–24 years) valued a service with HCT and ART (compared to a service offering HCT) less than younger (aged 15–19 years) respondents ( *β = −*0.285) and individuals currently in a relationship valued these additional service components more than those who are not in a relationship (*β =* 0.416).

The interaction terms also suggest that female respondents may be less interested in a service offering sports and recreation compared to male respondents (*β = −*0.424). Additional youth friendly components such as sports and health talks may contribute to creating a positive atmosphere for youth. However, the interaction term shows that the positive value that all respondents placed on this element is nearly halved when considering females only. Finally, individuals currently in a relationship are less sensitive to price (*β =* 0.002) than single respondents (population mean *β = −*0.006).

The standard deviations for all but one of the service attribute parameters are statistically significant indicating that there is substantial variation in preferences which cannot be explained by the observable characteristics included in the model. The very small weighting parameter γ suggests that the model is approaching a GMNL-II model in which the scale parameter impacts on both the means and the standard deviations of parameter estimates proportionately. The large and significant scale parameter (*p* < 0.01) confirms that scale heterogeneity is present among respondents indicating that the choice behaviour of some respondents may have been characterised by a greater degree of randomness (or uncertainty). It is also possible that respondents employed decision rules which resulted in near lexicographic preferences.

### Willingness to pay for service attributes

The analysis in WTP-space provides estimates of WTP for each service attribute. The results of the main analysis provided in Table 4 are expressed in Malawi Kwacha. Values are presented in 2011 USD in Table 5. The highest estimate of WTP for an attribute is associated with confidentiality with respondents estimated to be willing to pay USD$1.76 (p < 0.001) for a confidential service. This is consistent with the results of models estimated in utility space which all showed this to be the most important service attribute. This is followed by USD$0.65 (p < 0.001) for a service with both HIV testing and treatment available, USD$0.26 (p < 0.01) for a service including sports for young people. Estimates of WTP for remaining attributes are not statistically significant; however, they are broadly consistent with the results of the models estimated in preference space indicating that respondents are likely to value the addition of health talks and the availability of a younger service provider. The negative coefficient for provider gender suggests that on average respondents may value a male service provider which is inconsistent with previous models but the coefficient for this attribute was not statistically significant in this or any of the model specifications.

### Preferences for simulated service packages

**Figure 2:**
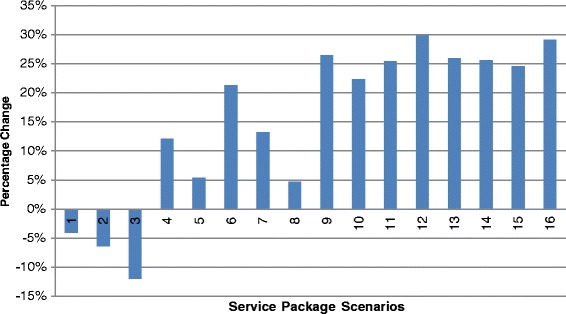
Proportion of Respondents Preferring Specified Scenario to Base Scenario.

The probability analysis shows the impact of a change in individual service attributes and the proportion of individuals expected to choose each simulated service package. A description of each of the simulated scenarios and relevant base case comparators is provided in Table 6. Changes in the choice probabilities associated with each of the simulated scenarios are shown in Table 7. Scenarios one through three in Figure 2 show the decreased proportion of respondents expected to choose a given alternative as price increases. At the highest price level of 500MK (approximately USD$2.00 at the time of the survey) up to 11.9% more of the sampled respondents would prefer the base case scenario of a service with a fee of 150MK. Scenarios four through eight show the impact of a change in only one service attribute. Scenarios four and five show that the addition of sports and recreation is likely to be more attractive than the addition of a health talk (increases of 12.1% and 5.4% of respondents are expected to choose packages with these elements respectively). Scenario six confirms the importance of confidentiality; the addition of this attribute shows the largest impact on preferences with 21.3% more respondents expected to choose the alternative with a confidential service compared to a service where clients may have concerns about confidentiality. Scenario seven shows that the availability of both HCT and ART and is preferred by 13.2% more clients than a service with HCT alone. Scenario eight shows that the age of the service provider is likely to have only a modest impact on preferences for a service package, with a 5% change in proportion of respondents preferring a service with a younger service provider

.

The combined service package scenarios indicate that the largest proportion of respondents are expected to prefer scenario 12 (4, 6 and 7) with up to 29.8% more respondents expected to choose a package of free, confidential services, with HCT and ART available and sports for young people. When the price of this package is increased to 50MK in scenario 16 (1, 4, 5 and 6), the increase in the number of respondents choosing this alternative compared to the base case is 29.1%. Interestingly, this smaller increase in respondents preferring this scenario is less than the decrease associated with only a 50MK increase in price (scenario 1), indicating that respondents trade between price and the additional service elements available in the package Figure 2.

**Figure 3:**
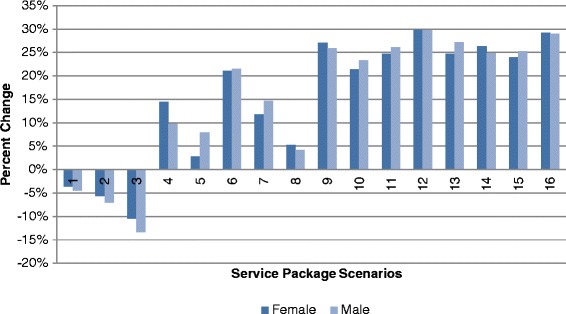
Proportion of Respondents Preferring Specified Scenario to Base Scenario According to Gender.

Separate analyses reveal variation in preferences for scenarios according to gender, age and relationship status (Table 7). These results show broadly similar preferences among male and female respondents for the service packages described in scenarios 12 and 16 (Figure 3)

**Figure 4:**
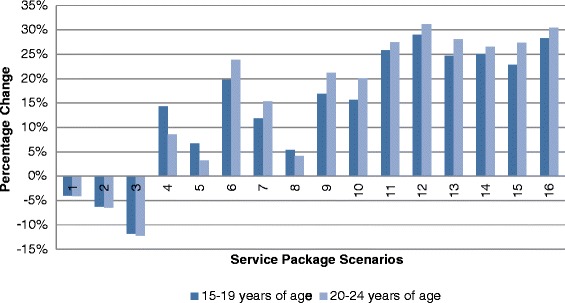
Proportion of Respondents Preferring Specified Scenario to Base Scenario According to Age.

. Simulations by age categories indicate that a higher proportion of respondents aged 20–24 prefer all simulated scenarios compared to base case scenarios. Again, the increase in the proportion of respondents preferring a simulated service package was highest in scenario 12 (31.1% of respondents aged 20–24 compared to 28.9% for respondents aged 15–19). This was followed by scenario 16 where the proportion of respondents preferring this scenario was 30.5% higher for older respondents and 28.2% for younger respondents (Figure 4)

**Figure 5:**
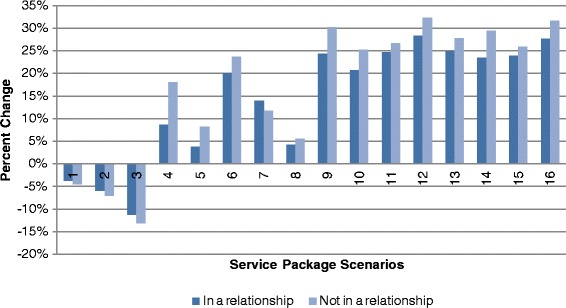
Proportion of Respondents Preferring Specified Scenario to Base Scenario According to Relationship Status.

. The largest differential appears in the analyses according to relationship status where the change in proportion of respondents expected to choose a simulated service package was highest for respondents who are not currently in a relationship across all scenarios (Figure 5)

.

## Discussion

The results of the analyses presented here suggest that when it comes to FP services, respondents value confidentiality more than any other service attribute. This result is not surprising and is consistent with the qualitative work done in developing this DCE and the literature available on youth preferences for SRH services [45,46]. In this setting confidentiality was described as a service where clients would feel confident that no one would overhear their consultation and the service provider would not divulge the details of the visit to anyone else. This definition covers concepts of privacy and secrecy but does not cover anonymity which is an element of confidentiality that young people may value [46], but which may be hard to ensure in an outreach setting.

Perhaps a more unexpected result is the importance of the HIV service package that is available in the context of FP service delivery. Respondents clearly value the availability of both HCT and ART services more than the presence of HCT alone. Given the low proportion of youth in this setting who are likely to be HIV positive and know their status, this suggests that youth valued these additional services regardless of whether they would actually use them. This suggests the value that youth place on this attribute may be more akin to an ‘option’ value [47] meaning that respondents place value on having the option to use this service, even though they may not use the service themselves. The positive value that youth place on the addition of ART to the HIV service package suggests that the availability of this service would not stigmatise the other services provided at the same time which can be a concern when considering SRH and HIV service integration [48]. This is supported by the findings from the qualitative work in which young people expressed concern that ART services should be readily available for those receiving a positive HIV test result.

The importance of the scale parameter clearly points to significant scale heterogeneity in responses. This type of heterogeneity cannot be explained by observable respondent characteristics and it is often unclear what the source is. Sivey *et al.* [49] have suggested that respondents who have difficulty in choosing, who had a hard time understanding the choice tasks or who have very low marginal utilities for some attributes may exhibit choice behaviour that appears to be nearly random which may result in a significant amount of heterogeneity in the scale parameter. Fiebig *et al.* [29] found this kind of behaviour to be more common in choice experiments with complex designs or those involving medical decision making. However, at present it is not possible to determine precisely which of these hypothesised sources of heterogeneity is driving this.

Given the unlabelled design of the DCE which presents two generic hypothetical situations and the framing of the choice tasks which asks respondents to imagine that they have already decided to access services, the results of the probability analysis and the proportion of respondents expected to choose a given service package are unlikely to correspond directly to increases in service utilisation. Simulated changes in probability also reflect in part the market simulated by the experimental design, which may not correspond directly to the actual market. As a result, these estimates are better understood as a measure of the acceptability of outreach services and may be used to understand which scenarios are most likely to be attractive or acceptable to young people.

Despite the significant amount of heterogeneity revealed in the model estimates, the results of the probability analysis consistently show that respondents prefer a service package that offers confidential services, both HCT and ART and sports for youth, with 29-32% of respondents expected to choose a service package including these three elements. Where service providers are unable to offer sports and HIV services, simulations indicate that 20% more respondents are expected to choose a confidential service compared to a service where clients may have concerns about confidentiality. This may offer some important insight to service providers aiming to improve the acceptability and youth-friendliness of their FP service provision with limited resources; where it is not possible to add additional service components, acceptability may still be improved by prioritising the elements of FP services that are most important to young people. This may be particularly important in areas where researchers have found that increasing the availability of youth-friendly SRH services is not sufficient to ensure that uptake increases among young people [8,9].

Estimates of WTP indicate that young people would be willing to pay for some service attributes. However, the results of the simulation analysis illustrate the impact of increasing price on young people’s preferences through the decreasing proportion of young people expected to choose a scenario decreases as price increases, with 12% fewer respondents expected to choose a service costing 500MK compared to a free service. While WTP is an informative measure and is helpful in terms of quantifying the relative importance of attributes, there are several reasons that it should be interpreted with caution in this setting. Firstly, within the survey sample the majority of respondents reported a monthly household income of USD $4 – 40, meaning that an estimated WTP of USD $1.76 for a confidential service could potentially be a large proportion of household income for many respondents. Secondly, while many young people in the sampled age range of 15–24 years may already be head of household and the majority of respondents indicated that they were engaged in some form of paid employment in the 12 months preceding the survey, many respondents in this age group would have been living with parents and many were not engaged in any form of employment. These respondents are unlikely to have direct access to their own financial resources and only indirect access to household resources. Many young people may therefore need to ask for financial resources to pay for services or for transportation to access services. Thirdly, young people in general, and particularly young women, may have limited capacity to make independent decisions about accessing FP services. Some young people may access services without their parents’ or partners’ knowledge, but making arrangements to attend services in secret may present a barrier to access for young people. Similarly, married women in this age category may be unable to make independent choices about contraceptive use, particularly in a cultural setting where child-bearing is highly valued and infertility stigmatised [28,29]. Finally, of 269 respondents who had previously accessed FP at either at a health facility or using an outreach service, over 80% reported receiving FP free of charge, indicating that very few respondents had experience in paying for FP services. As a result, drawing policy conclusions or pricing strategies based on estimates of WTP may prove problematic.

Overall, similarities in age and gender structure between the study sample and the general rural population in Malawi may mean that some results could be generalised to other rural settings in Malawi, particularly where analyses have revealed heterogeneity in preferences according to these characteristics. The relatively high reported rates of FP use among 15–19 year olds in the study sample may be a result of over reporting or it may mean that respondents in the study sample have better access to FP and face fewer barriers to access than young people in other areas of the country. If actual FP use is higher in the study population than the general population in rural Malawi then respondents may have more complete preferences and estimates of the proportion of respondents expected to choose specified service packages may be higher in this population than if similar work was completed with other groups. However, the results of this analysis did not indicate that previous FP use had a significant impact on preferences; indeed this was not included as an interaction term in the final model specifically because it was not a significant source of heterogeneity in any of the exploratory model specifications.

## Conclusion

Respondents completed a complex choice experiment and appeared to trade between outreach service attributes. There is considerable variation in young people’s preferences for FP service characteristics but targeting a few key areas of service design and delivery may result in similar improvements in service acceptability across all the sub-groups included in the analysis.

Further work could be done to understand the sources of heterogeneity and to determine how this may impact optimal service design and delivery strategies. This type of analysis may be of particular interest to policy makers interested in targeting a particular sub-group, such as girls or adolescents. This work could also be expanded to look at young people’s choice of SRH service provider in order to better understand preferences for outreach services compared to clinic-based services.

## Endnotes

^a^Respondents who do not have well-formed preferences may learn about their own preferences through the process of completing the choice tasks, or may construct preferences around the attributes presented in the choice tasks over time. These respondents are likely to be less consistent in their responses over time and this variation can show up as large or significant standard deviations around mean parameter estimates [50].

^b^In contrast to orthogonal experimental designs which aim to minimise correlation between attribute levels, efficient designs aim to produce data with as low as possible standard errors. Such designs incorporate prior parameter estimates to determine the asymptotic variance covariance (AVC) [51]. A d-efficient design is one where the determinant of the AVC is minimised. This is the most commonly used approach and the method used in both the pilot and final designs.

^c^This approach was used in order to create a design that could be used for either a forced choice or unforced choice format and analysed using an MNL or RPL approach. This involved specifying four utility functions in Ngene and using the ;model command to calculate the AVC matrix for each specification. Ngene then minimises a weighted average measure of efficiency across each model [51,52]. Maintaining this flexibility at the outset meant that both choice formats could be included in the questionnaire and participants who opted out could then be asked a follow-up forced choice question. In the event that many participants opted out this approach would allow for a complete forced choice dataset that could still be analysed. Since very few participants opted out in the final survey the unforced choice data are used for the present analysis.

^d^Guidance in the literature suggests that the first step in determining the sample size for a DCE is to determine the expected proportion of respondents choosing the least popular alternative. Using the unlabelled DCE with two alternatives (Service A and Service B) and an opt-out as a guide, it was estimated that roughly half of respondents would choose neither of the alternatives proposed (meaning they would choose the opt-out alternative) and the rest of the respondents will be equally split between the two. The second consideration is the number of choice sets to be completed by each respondent. Prior to the DCE development, it was anticipated that a maximum of 16 choice sets would be presented to each respondent in order to avoid overburdening respondents with too many choices. Finally, sub-group analyses were considered. In order to identify variation in preferences according to age and gender 4 sub-groups were identified; young women aged 15–19 years, young women aged 20–24 years, young men aged 15–19 years, young men aged 20–24. Based on these parameters, it was estimated that a minimum of 150 respondents for each of the sub-groups identified, or a total number of 600 (150 x 4) would be required. If each of the 600 respondents completed all 16 choice sets then 9600 observations (600 x 16) would be included in the data set. If 50% of the choices were ‘opt-outs’ then a remaining 4800 observations would still be available to analyse tradeoffs between the various attributes included in the service profiles. The 2010 DHS obtained a response rate of 98% and noted that response rates tended to be higher in rural areas [44]. Assuming that similar response rates could be achieved in the study sites, and assuming that each respondent would be able to complete the survey it was estimated that in order to obtain 600 completed surveys, approximately 620 individuals would need to participate in the survey.

^e^Effects coded variables use −1 as the base category instead of 0. For a variable with only two levels, this would be coded as −1 when the characteristic is not present and 1 when the characteristic is present. For three or more levels −1 remains the base category and the variable is coded as 1 when the characteristic present and 0 otherwise. This coding format allows effects to be separated from the overall mean effects measured in the utility function [22,53].

^f^Fiebig et al., [39] found that AIC correctly identified GMXL with correlated parameters as the true underlying model structure in 100% of model simulations.

^g^The Independence of Irrelevant Alternatives (IIA) assumption is not imposed in the RPL or GMXL framework as it is in an MNL model meaning that changes in probabilities associated with a change in more than one attribute are not a linear combination of changes estimated for individual attributes.
